# Beyond bacterial dysbiosis: the emerging role of gut bacteriophages in type 2 diabetes

**DOI:** 10.3389/fcell.2026.1735631

**Published:** 2026-08-24

**Authors:** Yimei Tao, Zhaoxiang Wang, Shao Zhong

**Affiliations:** 1 Gusu School, Nanjing Medical University, The First People’s Hospital of Kunshan, Kunshan, China; 2 Department of Endocrinology, Affiliated Kunshan Hospital of Jiangsu University, Kunshan, China

**Keywords:** bacteriophage, gut virome, metabolic inflammation, microbial metabolism, phage therapy, type 2 diabetes mellitus

## Abstract

Bacteriophages, the viruses that infect gut bacteria, are now seen as active members of the intestinal ecosystem rather than passive observers. In type 2 diabetes mellitus (T2DM), growing evidence suggests that the gut phage community changes in several important ways. People with T2DM often have lower phage diversity, more temperate phages, and shifts in bacterial hosts. These changes may spread through the microbial network, affecting gene exchange, bacterial metabolism, and immune activity. Through these pathways, phages may contribute to microbial and immune alterations associated with insulin resistance and the chronic inflammation that characterizes T2DM, even though direct proof of causality is still missing. Diet and metabolic stress also influence how phages behave, including their replication cycles and host preferences. This means that environmental factors can constantly reshape the gut virome and, in turn, affect metabolic health. Recognizing this link moves the focus beyond bacterial imbalance to a broader concept of phage-mediated metabolic dysregulation, in which viruses may represent underrecognized contributors to energy balance and inflammation. Understanding these interactions may reveal new microbial targets and guide the development of phage-based or microbiome-based approaches for the prevention and treatment of T2DM.

## 1 Introduction

Type 2 diabetes mellitus (T2DM) is one of the most prevalent and costly metabolic disorders worldwide. Its incidence continues to rise, and patients are being diagnosed at younger ages, often accompanied by multiple complications and heavy long-term medical burdens (Zheng et al., 2018). According to the latest data from the International Diabetes Federation (IDF), more than 500 million people are currently living with T2DM, and this number is expected to keep increasing in the coming decades (Schwarz, 2025). While traditional research has centered on insulin resistance, β-cell dysfunction, and genetic predisposition, these mechanisms alone cannot fully explain the complex relationships linking environmental exposure, lifestyle, and metabolic imbalance. This gap has prompted investigations into new biological pathways that may connect external factors with metabolic dysfunction.

In recent years, the gut microbiome has attracted growing attention as a crucial interface between environmental influences and host metabolism (Gilbert et al., 2018). It is now widely recognized that intestinal microbes play a key role in maintaining energy balance, modulating immune responses, and shaping disease risk. Numerous studies have shown that changes in bacterial composition, metabolic capacity, and microbial products can directly or indirectly influence insulin sensitivity, glucose homeostasis, and inflammation. In individuals with T2DM, the gut microbiota often shows a loss of diversity, a depletion of beneficial taxa, and an enrichment of opportunistic or pro-inflammatory species, all of which point to a close link between microbial dysbiosis and metabolic disturbance (Baars et al., 2024). These findings have reshaped the traditional view of T2DM, suggesting that the disease may also arise from an ecological imbalance within the gut.

However, the stability of the gut ecosystem depends on more than bacteria alone. The gut virome, especially bacteriophages, the viruses that infect bacteria, has emerged as an essential yet often overlooked component of this microbial network (Kurilovich and Geva-Zatorsky, 2025). Once regarded as passive background noise, phages are now seen as active participants that influence bacterial evolution and host metabolism. They interact with bacteria in both lytic and lysogenic cycles, altering microbial abundance, gene expression, and metabolic output. Through these processes, phages can shape the balance between health and disease at multiple levels. Experimental evidence supports this notion: fecal virome transplantation (FVT) from lean donors improves obesity and glucose intolerance in mice (Rasmussen et al., 2020), while similar interventions in patients with metabolic syndrome (MetS) lead to measurable changes in gut phage composition and metabolic indicators (Wortelboer et al., 2023). Such results suggest that the virome, rather than being a silent bystander, may actually help drive or mitigate metabolic dysfunction.

Rapid progress in viromics and multi-omics technologies is now beginning to uncover how bacteriophages participate in key metabolic pathways, including energy metabolism, bile acid signaling, and immune regulation. Yet, a large proportion of viral genomes within the human gut virome remain functionally unannotated, leaving the role of bacteriophages in metabolic homeostasis largely within the realm of “viral dark matter” (Aggarwala et al., 2017; Shkoporov and Hill, 2019). Most existing studies are still descriptive, focusing on shifts in community composition rather than mechanistic evidence or causality. This lack of functional insight raises an important question: through what interactions with bacteria and the host do phages contribute to the onset and progression of T2DM?

To address this question, the present review synthesizes current knowledge from ecological, functional, and translational perspectives. Relevant literature was identified through searches of PubMed and Web of Science using combinations of terms including “gut phageome”, “bacteriophage”, “gut virome”, “type 2 diabetes”, “metabolic syndrome”, “obesity”, “fecal virome transplantation”, and “phage therapy”. Studies were selected based on their relevance to gut bacteriophages and metabolic disorders, with priority given to original research articles, mechanistic studies, clinical investigations, and high-quality reviews published in English. Conference abstracts, non-peer-reviewed reports, and studies not directly related to gut phages or metabolic diseases were generally excluded. The detailed screening process is illustrated in Supplementary Figure 1. This review first outlines the baseline characteristics of the healthy gut phageome, then examines structural and functional alterations observed in T2DM, and finally discusses emerging strategies that target phage activity for metabolic regulation and therapy. By integrating evidence from metagenomics, experimental models, and interventional studies, this review aims to clarify how phage-associated metabolic dysregulation contributes to the onset and progression of T2DM and how these insights may guide the development of microbiome-based therapeutic approaches.

## 2 The baseline landscape of the healthy human gut phageome

The human gut hosts one of the densest and most complex viral ecosystems on Earth, containing roughly 10^9^–10^10^ viral particles per Gram of feces (Shkoporov and Hill, 2019; Rohwer, 2003). A large meta-analysis of more than 2,700 human gut metagenomes revealed that over 97% of these viral populations are bacteriophages, while eukaryotic and archaeal viruses account for only a small minority (Gregory et al., 2020). In healthy individuals, the gut phageome follows a characteristic “core–specific” architecture. The core gut phageome is commonly characterized by the predominance of crAss-like phages together with diverse tailed phages belonging to the Siphoviridae and Microviridae families (Shkoporov and Hill, 2019), is widely shared and persists over time, acting as an ecological backbone that stabilizes microbial networks (Manrique et al., 2016). In parallel, individual-specific phages vary across hosts and are shaped by diet, environment, and host genetics, forming unique “viral fingerprints” (Mahmud et al., 2024). This dual organization ensures both resilience at the community level and individuality at the person level. Longitudinal observations show that, despite short-term dietary shifts, the overall phage composition within an individual remains remarkably stable over years (Shkoporov and Hill, 2019). Such persistence reflects a strong self-regulating capacity of the virus–bacteria system and underlines its central role in maintaining gut homeostasis.

Spatial organization adds another layer of complexity. Phage abundance and diversity peak in the gut lumen, whereas the mucus layer, although less populated, is dominated by stable core phages such as *crAss-like* types (Yan et al., 2023). This vertical stratification shapes which bacteria can approach the epithelium, influencing mucosal immunity and barrier integrity. Along the intestinal axis, phage richness increases from the small intestine to the distal colon, likely reflecting spatial variation in bacterial density, oxygen availability, pH, and bile acid composition within the intestinal environment (Wu et al., 2024; Donaldson et al., 2016). Disrupting these structures, as observed in metabolic disorders such as T2DM, may weaken ecological resilience and promote chronic low-grade inflammation. These features together outline the ecological organization of a healthy gut virome and form the basis for understanding how viral networks help maintain microbial and metabolic balance.

### 2.1 Ecological dynamics and life-cycle balance

Beyond their abundance and structural diversity, bacteriophages play an active and nuanced role in shaping gut microbial ecology, the interconnected system linking bacteria, phages, and the host intestinal environment, through their life-cycle strategies (Donaldson et al., 2016). Based on their replication behavior, phages are traditionally categorized as lytic or temperate, although additional states such as chronic infection (Naureen et al., 2020) and pseudolysogeny (Łoś and Węgrzyn, 2012) may also occur under specific environmental conditions. Lytic phages regulate bacterial population dynamics by selectively lysing dominant species, a process known as the “kill-the-winner” effect (Winter et al., 2010), which helps maintain microbial diversity and ecological balance. Temperate phages, on the other hand, integrate into bacterial chromosomes as prophages, mediating horizontal gene transfer (HGT) and potentially carrying auxiliary metabolic genes (AMGs), antibiotic resistance determinants, or virulence factors that can reshape bacterial metabolism and influence host–microbe interactions (Mahmud et al., 2024). However, it should be noted that AMG identification in metagenomic datasets is still largely based on sequence homology and bioinformatic prediction, which may introduce false-positive annotations due to bacterial contamination, incomplete viral genome reconstruction, or uncertain functional assignment (Shkoporov and Hill, 2019). When exposed to stress conditions such as oxidative damage or shifts in pH, these prophages can be induced to enter the lytic cycle, thereby altering local microbial composition (Dieppa-Colón et al., 2025). Chronic infection, in which phages continuously release progeny without lysing the host cell, and pseudolysogeny (Zhang et al., 2025), a transient state associated with nutrient limitation or host stress, are thought to provide additional ecological flexibility under unstable environmental conditions (Venturini et al., 2022; Sutton and Hill, 2019). These non-canonical states may allow phages to persist within bacterial hosts without immediate lysis or stable genomic integration, thereby contributing to ecological adaptability within the fluctuating gut environment.

Remarkably, it is estimated that nearly 80% of gut bacterial genomes harbor prophage elements (Silpe et al., 2023), highlighting their ubiquity and their essential contribution to ecological resilience. Under physiological conditions, lytic and temperate phages coexist in a dynamic equilibrium characterized by controlled bacterial turnover, stable prophage induction rates, and preservation of core metabolic functions, balancing the opposing forces of “Piggyback the Winner” and “Kill the Winner” (Silveira and Rohwer, 2016). This reciprocal regulation helps prevent excessive expansion or collapse of dominant bacterial populations while maintaining ecological resilience within the gut microbiome, thereby supporting long-term intestinal homeostasis. When transient perturbations such as oxidative stress or nutrient limitation trigger prophage induction, the ecosystem often self-regulates and returns to equilibrium. However, under metabolic stress or chronic inflammation, this delicate balance may collapse, potentially initiating microbial dysbiosis and downstream host dysfunction.

### 2.2 Functional interactions and immune regulation

**FIGURE 1:**
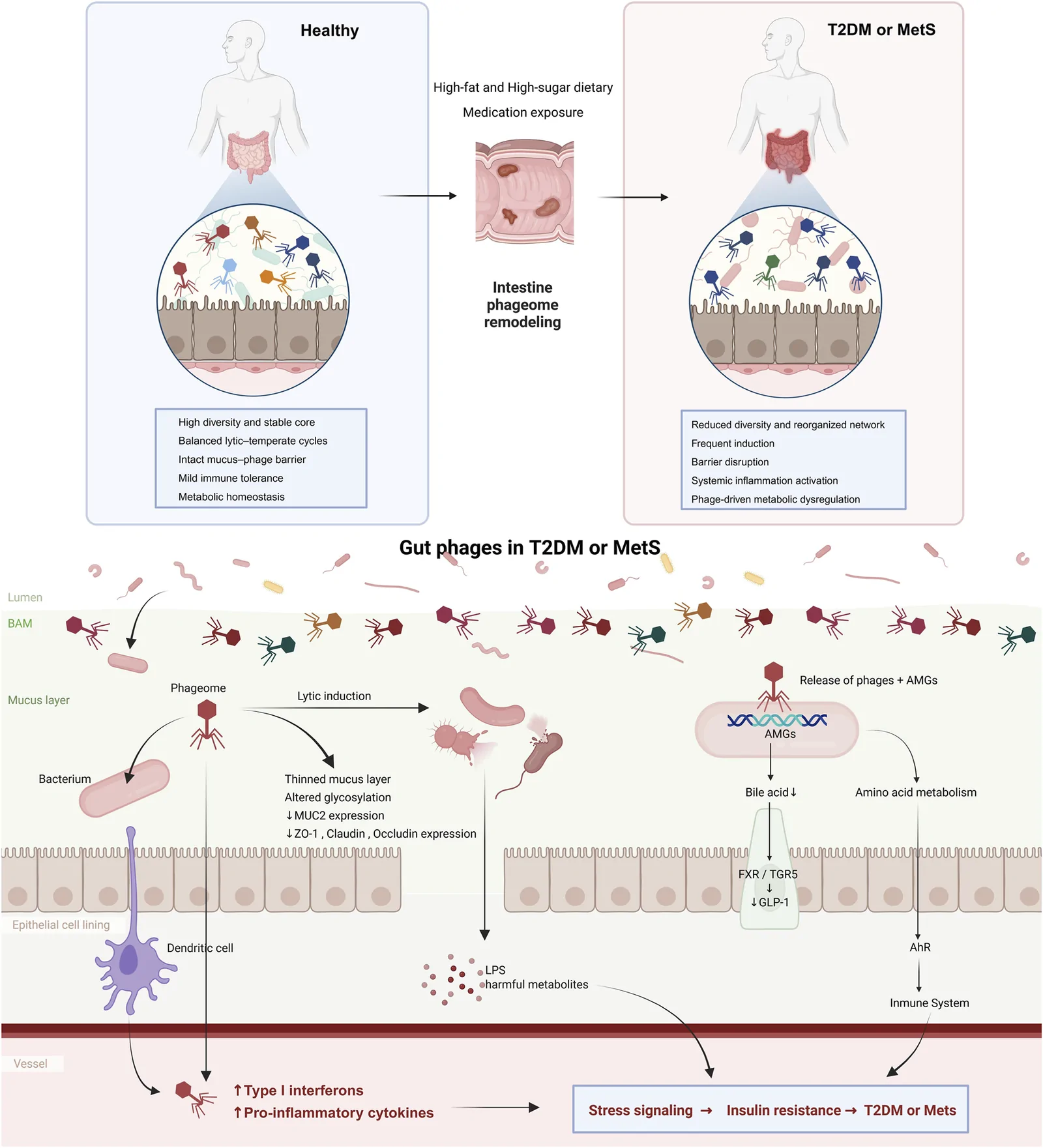
Integrated model of gut phageome remodeling and phage-mediated metabolic dysfunction in T2DM or MetS. This figure summarizes the proposed ecological, functional, and immunological mechanisms linking gut phage alterations to metabolic dysfunction in type 2 diabetes mellitus (T2DM) or metabolic syndrome (MetS). Under healthy conditions, lytic and temperate phages remain in ecological balance, and the bacteriophage adherence to mucus (BAM) layer contributes to mucosal protection and microbial stability. In T2DM and related metabolic disorders, reduced phage diversity, expansion of temperate phages, and altered phage–host interactions are associated with microbial dysbiosis and barrier dysfunction. Metabolic and oxidative stress may promote prophage induction, bacterial lysis, and lipopolysaccharide (LPS) release, thereby amplifying inflammatory signaling through TLR4/NF-κB pathways. Phage-mediated horizontal gene transfer (HGT) and auxiliary metabolic genes (AMGs) may further reshape bile acid metabolism, short-chain fatty acid signaling, and bacterial stress responses. Increased intestinal permeability may also allow phage particles or nucleic acids to access immune compartments and activate TLR9 and cGAS–STING pathways, contributing to chronic low-grade inflammation and insulin resistance. The depicted mechanisms are synthesized from metagenomic, in vitro, and animal studies; direct causal relationships in human T2DM remain to be fully established. T2DM, type 2 diabetes mellitus; MetS, metabolic syndrome; BAM, bacteriophage adherence to mucus; LPS, lipopolysaccharide; HGT, horizontal gene transfer; AMGs, auxiliary metabolic genes; TLR4, Toll-like receptor 4; NF-κB, nuclear factor kappa B; TLR9, Toll-like receptor 9; cGAS, cyclic GMP–AMP synthase; STING, stimulator of interferon genes; SCFA, short-chain fatty acid. Created in https://BioRender.com.

Beyond acting as bacterial predators, bacteriophages play an important role in keeping the gut environment stable. In healthy people, they are part of a layered network that connects the host, bacteria, and viruses through gene exchange and immune control (Guerin and Hill, 2020). On one hand, phages can reshape bacterial metabolism through HGT and by carrying AMGs. By adding new functional genes, phages can expand the metabolic capabilities of bacterial hosts and reshape microbial community function; however, at the same time, phage-mediated horizontal gene transfer may also promote the spread of virulence-associated genes, antibiotic resistance determinants, and other potentially harmful traits, highlighting the dual ecological role of phages within the gut environment. In fact, phages have been found to affect bacterial pathways related to carbohydrate, amino acid, and lipid metabolism (Mahmud et al., 2024). Through these effects, they can indirectly influence host energy balance and the production of short-chain fatty acids (SCFAs). This virus-driven modulation of microbial metabolism may contribute to microbial diversity and metabolic adaptability under healthy conditions, although similar mechanisms can also facilitate the spread of virulence-associated or pro-inflammatory traits under dysbiotic states. It also provides a useful model for understanding how these processes may become dysregulated in T2DM. On the other hand, phages communicate closely with the host immune system and help preserve mucosal balance. Some of them can attach to the intestinal mucus layer by binding their immunoglobulin-like proteins to mucin molecules, creating a physical barrier known as the bacteriophage adherence to mucus model, or BAM (Figure 1) (Barr et al., 2013; Barr et al., 2015). This layer limits the contact of harmful bacteria with epithelial cells and helps reduce inflammation. Phage DNA can also be detected by immune receptors such as TLR9 or the cGAS–STING pathway, causing a mild immune response and the production of low levels of neutralizing antibodies (Focà et al., 2015). Such gentle immune activity prevents infection while avoiding excessive inflammation, forming an essential part of gut immune tolerance. Early exposure to diverse phages may further shape this tolerance and support the normal development of the immune system (Dalmasso et al., 2014; Slavik and Kranzusch, 2023). However, once this balance is disturbed, as often happens in metabolic disorders like T2DM, the same sensing pathways may shift from protective regulation to stronger inflammatory signaling. This change may contribute to chronic inflammatory signaling associated with disease progression.

Phages contribute to gut ecosystem dynamics through gene exchange and immune modulation; however, phage-mediated horizontal gene transfer may also facilitate the dissemination of virulence or other deleterious factors. Once this balance breaks down, they may shift from stabilizers to triggers of inflammation and metabolic disruption, a process that likely marks the early stage of virome changes in T2DM.

## 3 Gut phage alterations and mechanistic links in T2DM

Under physiological conditions, lytic and temperate phages coexist in a dynamic ecological equilibrium characterized by controlled bacterial turnover, stable prophage induction rates, maintenance of microbial diversity, and preservation of core metabolic functions. However, in metabolic disorders such as T2DM, this delicate relationship begins to break down. Recent studies suggest that T2DM involves not only bacterial imbalance but also a more subtle and complex change in the phage population. Indeed, several characteristic patterns have been observed. Phage diversity tends to decline (Fan et al., 2023). The spatial structure within the gut barrier weakens, functional genes are reprogrammed, and immune activity rises. These shifts do not occur in isolation. Together, these alterations may represent interconnected ecological changes associated with systemic metabolic inflammation (Figure 1). In the sections that follow, we look at this process from three main angles: ecological change, gene transfer, and immune activation. These perspectives together clarify how phage activity may participate in the development and progression of T2DM (Figure 1).

### 3.1 Ecological and spatial remodeling

#### 3.1.1 Community composition and life-cycle patterns

Recent advances in metagenomics and virome sequencing have shown that the gut phage ecosystem in people with T2DM changes in several ways. These include shifts in community structure, life-cycle balance, and spatial organization. As summarized in Table 1, many studies have reported a reduction in phage α-diversity in T2DM, although these findings may also be influenced by dietary patterns, obesity, medication exposure, and other host-related factors (Yang et al., 2021). At the same time, β-diversity analyses show a clear separation between patients and healthy individuals, pointing to a broad reshaping of the phage community. Host prediction studies also reveal a change in host range (Yang et al., 2021; Chen et al., 2020). Phages that infect members of the Enterobacteriaceae and Bacteroidota families are often increased in T2DM, while those that target helpful bacteria such as Lactococcus and Bifidobacterium tend to decrease (Chen et al., 2020). These trends mirror bacterial dysbiosis and reflect the close ecological coupling between bacteria and their phages. Similar results have been found in people with MetS. For example, a study of 196 participants showed lower phage richness and diversity, along with more phages infecting Streptococcaceae and Bacteroidaceae, and fewer that target Bifidobacteriaceae (de Jonge et al., 2022). This pattern fits the “kill-the-winner” concept, suggesting that metabolic stress may disturb the balance between bacteria and their phages and weaken the normal ecological stability of the gut virome.

**TABLE 1:** Representative studies on gut phageome alterations in type 2 diabetes and metabolic disorders.

Study (year, journal)	Design/population (n)	Key methods	Main findings	Limitations
Yang et al. (2021) (2021, Gastroenterology)	Cross-sectional case-control; Chinese adults with obesity/T2DM and lean controls (n = 229)	Fecal VLP isolation, shotgun metagenomics, virome-bacteriome analysis	Obesity and T2DM were associated with reduced gut viral diversity and weakened transkingdom interactions	MDA amplification bias; RNA viruses not assessed
Chen et al. (2020) (2020, Front. Cell. Infect. Microbiol.)	Cross-sectional case-control; T2D vs controls (n = 46)	VLP metagenomics, 16S sequencing, inflammatory marker analysis	T2D showed altered Enterobacteriaceae-associated phages and elevated LPS levels; phage signature discriminated T2D	No external validation; medication confounding; MDA bias
Fan et al. (2023) (2023, Gut Microbes)	Cross-sectional case-control; T2D with/without diabetic nephropathy and controls (n = 132)	VLP sequencing, 16S sequencing, XGBoost diagnostic modeling	Viral diversity and phage-host interactions were reduced in T2D/DN; combined viral-bacterial markers showed high diagnostic accuracy	Cross-sectional design; lack of external validation; lifestyle confounding
de Jonge et al. (2022) (2022, Nature Communications)	Cross-sectional case-control; MetS vs controls (n = 196)	WGS metagenomics, VLP sequencing subset, viral clustering and host prediction	MetS associated with lower virome diversity and altered phage composition; identified Ca. Heliusviridae lineage	No causal inference; low host assignment rate; lack of viral isolation
Manrique et al. (2016) (2016, PNAS)	Longitudinal and cross-cohort observational study	Deep virome sequencing, network analysis	Defined a shared “healthy gut phageome” reduced in intestinal diseases	Very small primary cohort; fragmented viral assemblies
Wortelboer et al. (2023) (2023, Nature Communications)	Double-blind randomized placebo-controlled trial; MetS patients (n = 24)	Sterile fecal filtrate transfer (FFT), OGTT, longitudinal virome metagenomics	FFT transiently reshaped gut phage communities but did not improve glycemic outcomes	Small sample size; transient effect; non-phage components may confound
Rasmussen et al. (2020) (2020, Gut.)	Randomized mouse experiment; diet-induced obesity model (n = 40)	Fecal virome transplantation (FVT), OGTT, metagenomics, metabolomics	FVT from lean donors improved glucose tolerance and reduced weight gain in obese mice	No obese-donor FVT control; cage effects; MDA bias
Mao et al. (2024) (2024, Nature Communications)	Randomized mouse experiment; HFD-induced MetS (n = 48)	Modified FVT strategies, virome sequencing, immune profiling	Optimized virome transfer improved glucose regulation and altered gut microbial networks	Small sample size; heterogeneous response; incomplete removal of eukaryotic viruses
Ye et al. (2023/12) (2023, Appl. Microbiol. Biotechnol.)	T2D mouse model; HFD/STZ-induced diabetes (n = 30)	Oral phage cocktail therapy, OGTT, 16S sequencing, inflammatory assays	Phage treatment improved insulin resistance, restored SCFA-producing bacteria, and reduced inflammation	Direct phage targets unclear; mechanistic causality unresolved
Oh et al. (2019) (2019, Cell Host & Microbe)	Dietary crossover mouse study (n = 24)	Prophage induction assays, bacterial genetic knockouts, metabolite analysis	High-fructose diet and SCFAs induced prophage activation through Ack/SOS pathways	Mechanistic details incomplete; limited translation to humans
Ma et al. (2018) (2018, Microbiome)	Retrospective multi-cohort case-control; T2D vs controls (n = 370)	Public WCMS data mining, phage scaffold identification, network analysis	Identified T2D-specific phage signatures and altered phage-bacteria interaction networks	Purely computational; no experimental validation; poor ssDNA/RNA virus coverage
Scheithauer et al. (2024) (2024, Gut Microbes.)	Exploratory case-control; T2D donors and healthy controls	Purified fecal phage stimulation of human DC/T-cell co-culture, metagenomics	T2D-derived phages induced stronger inflammatory immune responses and IFN-γ production	Small sample size; residual contaminant risk; exploratory *in vitro* design
Campbell et al. (2020) (2020, Cell Reports)	Germ-free mouse and *in vitro* mechanistic study (n = 7 mice)	Phage-host genetic engineering, RNA-seq, bile acid metabolomics	Phage BV01 altered bile acid metabolism via repression of bacterial tspO expression	Small sample size; limited *in vivo* validation
Gogokhia et al. (2019) (2019, Cell Host Microbe.)	Mouse models and human UC cohorts	Purified phage stimulation, immune profiling, RNA-seq	Phages directly activated TLR9–IFN-γ inflammatory pathways and aggravated colitis	Complex *in vivo* immune networks not fully resolved

This table summarizes representative human, animal, and in vitro studies investigating the associations between the gut phageome and type 2 diabetes mellitus (T2DM), obesity, metabolic syndrome (MetS), and related metabolic complications. The included studies encompass observational cohorts, randomized intervention studies, mechanistic animal experiments, and cell-based investigations. Major methodologies included virus-like particle (VLP) enrichment, shotgun metagenomic sequencing, fecal virome transplantation (FVT), immune profiling, metabolomics, and phage-host interaction analyses. Overall, these studies suggest that metabolic disorders are frequently associated with reduced gut viral diversity, altered bacteriophage composition, disrupted phage–bacteria interaction networks, and enhanced inflammatory signaling. Experimental studies further indicate that modulation of the gut phageome may influence glucose metabolism, insulin resistance, microbial ecology, and host immune responses. The limitations listed for each study highlight current methodological challenges in gut virome research, including amplification bias, limited viral host assignment, insufficient external validation, small sample sizes, and the lack of causal or mechanistic confirmation.

Abbreviations: T2DM, type 2 diabetes mellitus; MetS, metabolic syndrome; DN, diabetic nephropathy; VLP, virus-like particle; WGS, whole-genome shotgun sequencing; FFT, fecal filtrate transfer; FVT, fecal virome transplantation; OGTT, oral glucose tolerance test; HFD, high-fat diet; STZ, streptozotocin; SCFA, short-chain fatty acid; LPS, lipopolysaccharide; WCMS, whole-community metagenomic sequencing; DC, dendritic cell; IFN-γ, interferon gamma; UC, ulcerative colitis.

At the compositional level, several phage groups have been linked to metabolic problems. In a large human gut phage catalog, Ma and colleagues found specific phage operational taxonomic units enriched in T2DM samples (Ma et al., 2018). These changes could not be fully explained by the abundance of their bacterial hosts, which suggests that phage–host relationships are being reorganized. Using virus-like particle sequencing, Chen and colleagues also observed more phages related to Enterobacteriaceae, including *Escherichia coli*, in T2DM patients (Chen et al., 2020). This finding indicates that phage–host pairs may shift under metabolic stress. Environmental factors such as inflammation, oxidative stress, and diet-related changes may further activate prophages, leading to bacterial lysis and the release of endotoxins. This “stress–lysis cycle” may undermine microbial stability. In fact, the abundance of certain phages has been linked to fasting glucose, insulin, and C-reactive protein levels (Chen et al., 2020). One study identified a panel of bacteriophages capable of distinguishing T2DM from healthy controls with an area under the receiver operating characteristic curve (AUC) exceeding 0.99; however, this figure should be interpreted with caution, as it derives from a single cohort without independent external validation and may reflect overfitting rather than true generalizability. To improve readability, the detailed phage list and their associated bacterial hosts are summarized in Table 2, though this performance has not yet been validated in independent cohorts, and may partly reflect cohort-specific overfitting (Table 2) (Fang and Ning, 2024). This strong predictive power suggests that these phages might serve as useful biomarkers for T2DM diagnosis or management. Overall, these findings indicate that the activation of temperate phages may act not only as a sign of ecological disturbance but also as a process that potentially influences metabolic imbalance.

**TABLE 2:** Phages reported to Be enriched in type 2 diabetes mellitus and their predicted bacterial hosts.

Phage/Phage group	Putative bacterial host	Alteration in T2DM	Potential significance
Enterobacteriaceae-specific phages	Enterobacteriaceae (e.g., *Klebsiella*, *Shigella*)	Increased	May contribute to elevated LPS release and metabolic inflammation
*Enterococcus* phage phiFL2A	*Enterococcus*	Increased	Included in the high-performance diagnostic phage panel
Brochothrix phage NF5	Brochothrix	Altered significantly	Identified as a discriminatory phage in T2DM
*Streptococcus* phage PH10	*Streptococcus*	Altered significantly	Contributed to T2DM classification model
*Streptococcus* phage 7201	*Streptococcus*	Altered significantly	Associated with T2DM-related phageome shifts
Streptococcus-associated phages (3-phage cluster)	*Streptococcus*	Selected by RF model	Important contributors to AUC >0.99 diagnostic model
Klebsiella-associated phages	*Klebsiella*	Increased	Enriched Enterobacteriaceae-related phages in T2DM
Shigella-associated phages	*Shigella*	Increased	Potentially linked to endotoxemia and inflammation

Representative bacteriophages were summarized based on differential abundance analysis and the random forest (RF) classification model reported by Chen et al. (2020). The study identified an eight-phage consortium capable of distinguishing T2DM patients from healthy controls with an AUC exceeding 0.99, although external validation was lacking.

Abbreviations: RF, random forest; T2DM, type 2 diabetes mellitus; AUC, area under the receiver operating characteristic curve.

Besides the loss of diversity, T2DM is also marked by a shift in host preference. Comparative studies show that phages from healthy donors mostly infect *Lactococcus* (Sutton and Hill, 2019), while those from T2DM individuals prefer *Bacteroides* (Ma et al., 2018). This shift points to a broader reorganization of the phage–bacteria network under metabolic stress. Since *Bacteroides* play an important role in energy metabolism, SCFA production, and immune regulation (Price et al., 2024), this redirection of phage targeting may interfere with microbial functions and promote immune activation. Phage changes in T2DM extend beyond abundance or structure, reflecting a shift in bacterial targets that may set the stage for metabolic inflammation.

#### 3.1.2 Spatial distribution and barrier function alterations

When structural changes in the gut phageome come with shifts in spatial organization, the stability of the mucosal barrier may become even more fragile. Under normal conditions, phages help maintain mucosal balance through their spatially organized interactions, especially through the so-called BAM barrier, which contributes to mucosal defense by creating a phage-enriched antimicrobial layer within mucus. As described by Barr (Barr et al., 2013; Barr, 2017), the BAM model suggests that some phages have immunoglobulin-like (Ig-like) domains that can bind to mucin glycoproteins such as MUC2. This allows them to attach to the mucus layer and create a protective “viral shield” (Almeida et al., 2019). Such a layer provides a physical defense that blocks pathogens from reaching epithelial cells before the immune system is even activated (Figure 1). Experimental studies based on mice have shown that phages like øPNJ-6, T4, and certain *E. coli* phages can bind to MUC2 through the Hoc protein and stimulate epithelial cells to produce more mucin (Wu et al., 2024). In this way, they help reduce colonization by enterotoxigenic and Shiga toxin-producing *E. coli*. Phage–mucus interactions can also promote goblet cell activity and enhance mucin secretion, forming a positive feedback loop that supports barrier integrity (Fang et al., 2021).

In metabolic disorders such as T2DM, this spatial organization becomes unstable. People with T2DM often have a thinner mucus layer, reduced mucin expression, and altered glycosylation patterns (Figure 1). At the same time, mucus-associated bacteria become imbalanced, and tight junction proteins like ZO-1, Claudin, and Occludin are expressed at lower levels (Dubois et al., 2023; Jatana et al., 2024; Siqueira et al., 2024). These changes alter the chemical properties of the mucus and its surface glycans, weakening the ability of phages to attach through their Ig-like domains. As a result, mucus-adherent phages decrease, the BAM barrier loses stability, and pathogens gain easier access to epithelial cells, which can trigger local inflammation. Once this spatial structure is disrupted, the consequences may go beyond the gut (Figure 1). During bacterial lysis, both phages and bacterial components such as lipopolysaccharides (LPS) can damage tight junctions and further increase intestinal permeability (Wei, 2024). This allows phages and microbial metabolites to enter the bloodstream, promoting systemic inflammation. Over time, this “barrier breakdown–phage translocation–inflammation” cycle may contribute to the chronic low-grade inflammation often seen in T2DM (Figure 1). So far, there have been few studies directly examining the BAM structure in metabolic diseases, which is a clear research gap. Still, existing evidence on mucus impairment suggests that metabolic stress may reshape phage spatial distribution by changing mucus composition, weakening the BAM-mediated defense in the process.

The gut phageome in T2DM displays reduced diversity, weakened spatial layering, and altered host preference. These structural shifts reveal changes in both viral stability and ecological function, forming the background for the following discussion on phage-associated genetic and metabolic reprogramming (Figure 1).

### 3.2 Functional gene transfer and metabolic signal reprogramming

In T2DM and related metabolic disorders, bacteriophages may not only reshape microbial community structure but also potentially drive deep functional remodeling of gene networks and metabolic pathways. This process, often described as phage-associated metabolic signal reprogramming (Borodovich et al., 2022), occurs when phages integrate AMGs or mediate HGT (Moura de Sousa et al., 2023; Luo et al., 2022), thereby endowing bacteria with new metabolic capabilities. Through these molecular exchanges, phages influence both microbial ecology and host metabolic homeostasis (Figure 1).

Temperate phages, which integrate into bacterial genomes as prophages, serve as key agents of functional reprogramming. Once integrated, prophages can carry or regulate a wide range of metabolic genes, including carbohydrate-active enzymes (CAZymes), amino acid metabolism enzymes, and bile acid transport-related genes, altering bacterial metabolic capacity and ecological behavior (Ji et al., 2025). In MetS, phages infecting *Bacteroides* are particularly enriched, suggesting close links between these phage–host interactions and host metabolism (de Jonge et al., 2022; Ma et al., 2018). For example, the *Bacteroides vulgatus* phage BV01 integrates into the *tspO* promoter region, suppressing genes involved in bile acid deconjugation and reducing secondary bile acid production (Campbell et al., 2020). This shift increases conjugated bile acids while decreasing secondary bile acids, altering signaling through FXR and TGR5 receptors (Kim and Fang, 2018). The result is excessive FXR activation and weakened TGR5 signaling, potentially leading to reduced GLP-1 secretion, lower energy expenditure, and enhanced lipid storage (Kim and Fang, 2018; Xie et al., 2021), highlighting how phages may help sustain metabolic inflammation through coordinated control of metabolite and immune signaling.

Metagenomic studies suggest that bacteriophages may play a role in the metabolic remodeling associated with T2DM by carrying various AMGs (Ma et al., 2018; Ji et al., 2025). These genes participate in carbohydrate, amino acid, and nucleotide metabolism (Luo et al., 2022; Avellaneda-Franco et al., 2023; Yan et al., 2025). Arginine-derived metabolites modulate nitric oxide (NO) production and vascular tone, while tryptophan metabolites activate the aryl hydrocarbon receptor (AhR) to regulate immune balance (Hezaveh et al., 2022). Thus, phages may help sustain metabolic inflammation through coordinated control of metabolite and immune signaling. In addition, certain prophage-associated genes such as htpG (Dong et al., 2021), which promote bacterial stress tolerance, may enhance bacterial survival in inflammatory environments and perpetuate metabolic imbalance. Beyond integration effects, phages in the lytic cycle can mediate gene transduction, facilitating HGT between bacteria. Experimental evolution studies in mice have shown that exogenous *E. coli* can acquire carbon utilization genes from resident microbiota via phage-mediated transfer, improving its metabolic fitness and competitive advantage (Frazão et al., 2019). Such phage-mediated gene flow allows the rapid spread of key functional genes, reconfiguring metabolic potential at the community level. In T2DM, this process may enable pathobionts to acquire genes involved in oxidative stress resistance, LPS biosynthesis, and amino acid decarboxylation, enhancing their pro-inflammatory potential and creating a self-reinforcing phage–microbiota–metabolism–immunity loop.

Insights from inflammatory bowel disease (IBD) research provide additional clues. In *Bacteroidales*, reversible DNA inversions drive phase variation that modulates polysaccharide and immune-related molecule synthesis. For instance, the promoter of polysaccharide A (PSA) in *Bacteroides fragilis* often switches to the “OFF” orientation in IBD patients, reducing anti-inflammatory PSA expression and lowering Treg and IL-10 levels. Certain phages were associated with enrichment of the “OFF” configuration in inflammatory settings, suggesting that phages may contribute to bacterial functional remodeling and inflammatory persistence (Carasso et al., 2024). Given the parallels between IBD and T2DM in chronic inflammation and phage alterations, raise the possibility that similar phase variation mechanisms could also occur in metabolic disease contexts, weakening microbial immune protection and potentially aggravating insulin resistance.

Despite metagenomic evidence supporting phage involvement in metabolic remodeling, our current understanding remains largely descriptive. Most datasets rely on sequence-based annotation, with limited functional validation linking phage genes to transcriptional activity, metabolic flux, or host outcomes. In other words, the field still operates on static catalogs rather than dynamic functional networks. Future research should integrate metagenomics with metatranscriptomics and metabolomics to establish causal relationships among phages, microbiota, and metabolites, ideally supported by controlled phage–host model systems to verify functional effects. Phages are increasingly recognized as potential contributors to microbial gene exchange and host–microbiota signaling within the gut ecosystem. Their dysregulation in T2DM may contribute to links between microbial functional disturbances and host metabolic dysfunction, highlighting their possible relevance as therapeutic targets.

### 3.3 Immune activation and inflammatory amplification

Under physiological conditions, bacteriophages contribute to mucosal homeostasis and immune tolerance through their interactions with both bacteria and the epithelial barrier. In metabolic disorders such as T2DM, however, this finely tuned balance appears to deteriorate. Accumulating data suggest that gut virome alterations, together with bacterial dysbiosis, impaired barrier integrity, and systemic metabolic stress, may collectively shift immune responses from a tolerant to an activated state. Although causal relationships remain to be firmly established, current evidence supports a conceptual framework in which phages participate in inflammatory amplification through both direct immune sensing and indirect modulation of bacterial activity.

Phages are increasingly recognized as potential mediators of host immune signaling. Decades ago, scientists proposed that mammalian immunity could recognize phages as either pro- or anti-inflammatory agents (Barr et al., 2013; Van Belleghem et al., 2017). Subsequent studies have provided supporting evidence. Gogokhia and colleagues reported that phages isolated from patients with inflammatory bowel disease (IBD) elicited stronger IFN-γ responses in murine models than those derived from healthy individuals (Scheithauer et al., 2024), suggesting that phage particles or their nucleic acids can be detected by immune cells and may influence cytokine production. Consistent with this view, phage DNA or virus-like particles have been identified not only in the intestinal lumen but also in blood, lymph, and other tissues (Van Belleghem et al., 2017). While the physiological implications of these findings remain uncertain, they raise the possibility that translocation of phages, or fragments of their genetic material, might occur when the intestinal barrier becomes compromised.

In people with T2DM, increased intestinal permeability, known as “leaky gut”, is a well-documented phenomenon (Snelson et al., 2021) (Figure 1). Under such conditions, it is plausible that phages or phage-infected bacteria could traverse the mucosal barrier, entering epithelial or immune compartments. Experimental studies indicate that phages can cross epithelial layers by transcytosis or through infected bacterial “Trojan horses” (Nguyen et al., 2017). Once within host tissues, phage DNA or capsid proteins may engage nucleic acid–sensing receptors such as TLR9 and the cGAS–STING pathway, leading to the release of type I interferons and other pro-inflammatory cytokines. Supporting this notion, Gogokhia et al. showed that *E. coli* and *Bacteroides* phages triggered IFN-γ production in a TLR9-dependent manner and exacerbated colitis (Gogokhia et al., 2019). Likewise, Achermann and colleagues observed that phage preparations derived from individuals with T2DM enhanced dendritic-cell activation and T-cell IFN-γ secretion *in vitro*, accompanied by increased CD86 expression (Scheithauer et al., 2024). Although direct *in vivo* confirmation in T2DM is still lacking, these results collectively suggest that phage components may act as immunostimulatory cues under metabolic stress.

Beyond direct immune sensing, phages may also modulate inflammation indirectly through their effects on bacterial hosts. In T2DM, members of the Enterobacteriaceae family and their associated temperate phages are often enriched (Chen et al., 2020). Metabolic and oxidative stress may promote prophage induction, causing bacterial lysis and the local release of lipopolysaccharide (LPS), which activates the TLR4/NF-κB pathway and stimulates cytokines such as TNF-α, IL-6, and IL-1β (Chen et al., 2020) (Figure 1). Although quantitative evidence remains limited, this process provides a plausible mechanistic link between phage activity, microbial instability, and low-grade inflammation. Moreover, during lysogeny, phages can transfer virulence or stress-response genes through HGT, enhancing bacterial adhesion or immune activation. Under inflammatory conditions, these HGT events may occur more frequently, forming a reinforcing loop in which phage induction, bacterial turnover, and host immune signaling perpetuate each other, a pattern reminiscent of inflammatory amplification described in IBD and MetS (Figure 1).

Not all immune interactions with phages are detrimental. Depending on the ecological and metabolic context, certain phages may exert protective or anti-inflammatory effects. Some phages can bind bacterial cells or directly neutralize LPS, thereby reducing its access to immune receptors and limiting reactive oxygen species production by phagocytes (Kurilovich and Geva-Zatorsky, 2025). Mucus-adherent phages, described under the BAM model, also form a non-cellular protective layer that reduces pathogen contact with the epithelium without provoking inflammation. These observations underscore the dual nature of phage–immune interactions: while they may amplify inflammation once immune tolerance is lost, they can also contribute to barrier defense and immune equilibrium under healthy conditions.

Taken together, the immunological role of phages in T2DM appears to be context dependent and mechanistically diverse. Current findings support a model in which metabolic stress and barrier dysfunction alter phage distribution and immune visibility, shifting their function from stabilizers to modulators, and, under persistent dysbiosis, to amplifiers of inflammation. Although the precise contribution of these processes to systemic insulin resistance remains to be clarified. It is equally important to recognize that systemic inflammation and intestinal permeability in T2DM are themselves strongly shaped by obesity, aging, and dietary patterns, making it challenging to attribute observed phage-immune interactions specifically to the diabetic state rather than to the broader metabolic milieu. Understanding the molecular dialogue between phages, bacteria, and host immunity could shed new light on the inflammatory underpinnings of metabolic disease and open avenues for targeted microbiome-based interventions (Figure 1).

## 4 Exogenous regulatory factors

### 4.1 Dietary stress and phage activation

The onset and progression of T2DM are shaped not only by genetic and metabolic factors but also by environmental influences, especially diet. Nutrients can reshape the gut metabolic microenvironment by altering substrate availability, redox balance, and levels of metabolic stress. These shifts not only reshape bacterial diversity and activity but also have a strong impact on phage life cycles and overall ecological stability. Growing evidence (Boling et al., 2020; Howard et al., 2024) suggests that diet-induced changes in phage behavior may act as a key link between environmental cues and host metabolic dysfunction, influencing both the risk and course of T2DM.

Recent studies show that many dietary components can directly affect prophage activation and replication, leading to major changes in the gut virome (Boling et al., 2020; Howard et al., 2024). In one large *in vitro* screening of 117 common foods and plant extracts, Boling and colleagues found that sweeteners, herbs, and flavoring agents could trigger phage activation in specific bacterial species (Boling et al., 2020). For example, stevia, propolis, and aspartame significantly increased the production of virus-like particles, while antimicrobial compounds such as N-acetylcysteine did not. This study provided the first experimental evidence that dietary molecules can reactivate prophages and reshape viral ecology, giving new insight into how diet, phages, and host metabolism are connected in conditions like T2DM.

**FIGURE 2:**
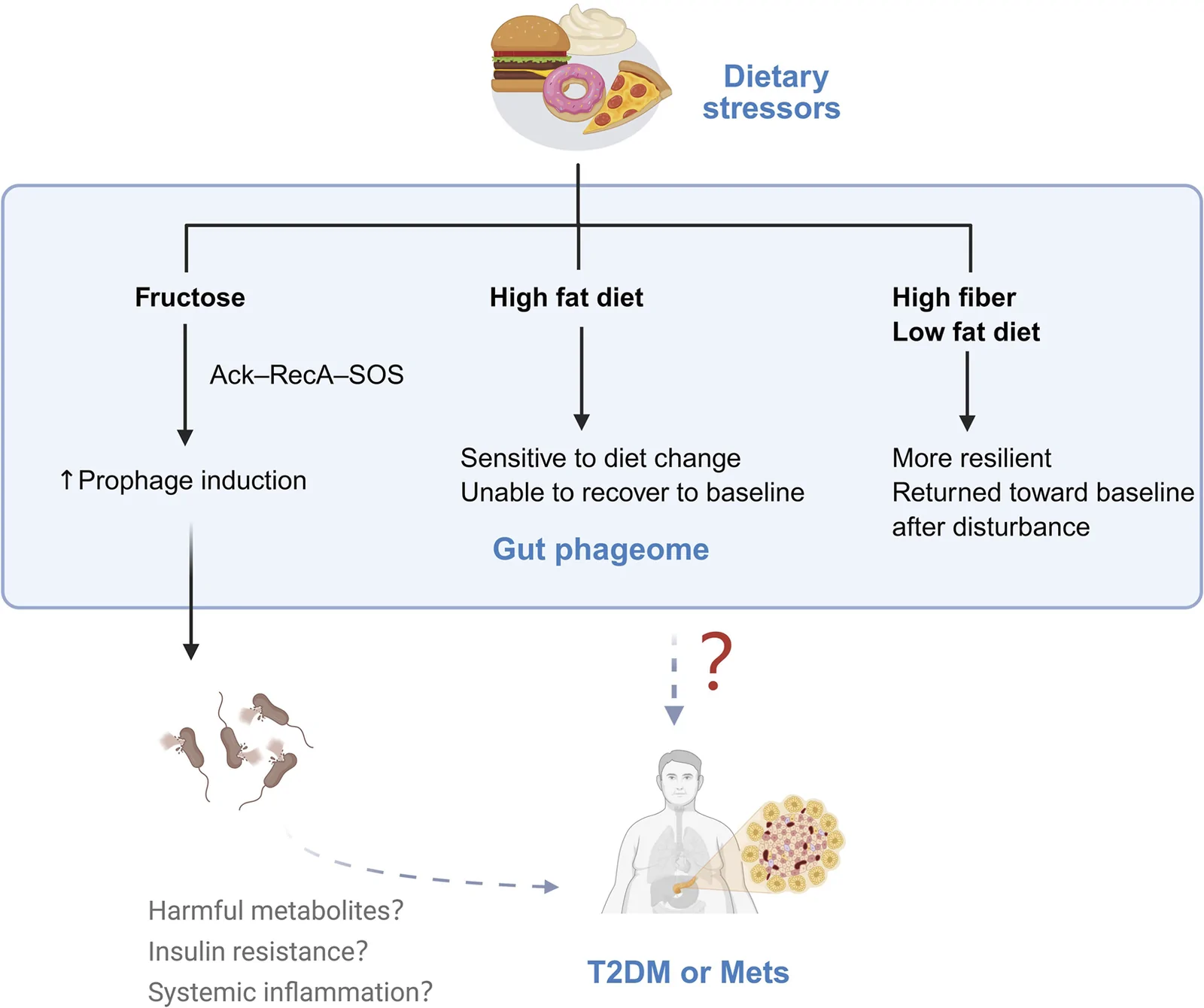
Dietary stressors influence gut phageome dynamics and their potential links to T2DM or Mets. Different dietary stressors can alter gut phageome composition and stability. Fructose intake may induce prophage activation through the Ack–RecA–SOS pathway, whereas high-fat diets increase phageome sensitivity and impair recovery after dietary perturbation. In contrast, high-fiber and low-fat diets are associated with greater phageome resilience. These diet-driven phageome alterations may contribute to harmful metabolite production, insulin resistance, systemic inflammation, and potentially the development of T2DM or metabolic syndrome (MetS). T2DM, type 2 diabetes mellitus; MetS, metabolic syndrome; Ack, acetate kinase; RecA, recombinase A; SOS, bacterial SOS response pathway. Created in https://BioRender.com.

While most research has focused on how diet alters bacterial communities, phages often show stronger and more persistent responses to dietary stress (Minot et al., 2011). In a dietary intervention comparing a high-fiber, low-fat diet with a Western-style diet rich in sugar and fat, the latter caused pronounced shifts in the gut virome, especially among bacteriophages, that did not return to baseline even after the diet stopped (Minot et al., 2011) (Figure 2). This lasting alteration suggests that high sugar intake may leave a long-term imprint on the virome, serving as an ecological “memory” of unhealthy diet exposure (Howe et al., 2016) (Figure 2). Unlike bacterial populations, which tend to recover after dietary improvement, the virome appears less resilient. Genomic analyses further revealed that these dietary changes were accompanied by an increase in temperate phages carrying genes related to integration, excision, and replication control (Kim and Bae, 2016). Many of these phages infect bacteria belonging to the *Clostridiales* and *Bacillales* orders, which are frequently enriched in individuals with T2DM (Fujimoto et al., 2022). These findings imply that diets high in fat and sugar may selectively activate temperate phages, pushing them from the lysogenic to the lytic cycle and thereby disturbing microbial balance through changes in bacterial composition and metabolism (Schulfer et al., 2020). Such phage-mediated ecological instability could represent an important mechanism contributing to the development of T2DM (Schulfer et al., 2020).

At the molecular level, experimental studies have begun to uncover how specific dietary metabolites can trigger these virome shifts through bacterial metabolism (Chatterjee and Duerkop, 2019; Oh et al., 2019). Many gut bacteria carry prophages that remain dormant under normal conditions but can be activated by metabolic or environmental stress (Chatterjee and Duerkop, 2019). Changes in nutrient composition can stimulate bacterial signaling pathways that lead to prophage excision and replication, disrupting the balance between lysis and lysogeny (Chatterjee and Duerkop, 2019; Oh et al., 2019). In a well-characterized *Lactobacillus reuteri* model, fructose and SCFAs acted as metabolic stressors that enhanced acetate metabolism through the enzyme AckA (Barr et al., 2013). This metabolic alteration led to the buildup of acetyl phosphate, caused DNA damage, and activated the RecA-dependent SOS response, which in turn triggered prophage induction (Chatterjee and Duerkop, 2019; Oh et al., 2019). Interestingly, fructose (Figure 2) selectively activated the LRɸ1 prophage, whereas SCFAs induced both LRɸ1 and LRɸ2, releasing large numbers of phage particles (Oh et al., 2019). These results suggest that certain dietary metabolites may directly affect phage life cycles in specific experimental contexts by altering bacterial metabolic activity, forming a mechanistic link between diet, bacteria, and phages that connects nutrient-driven metabolic stress with phage activation and gut microbial imbalance (Figure 2).

Taken together, diet affects gut phage dynamics through several pathways, including metabolic stress, signaling cascades, and microbe–microbe interactions. Diets rich in fat and refined sugar tend to promote a stress-prone virome dominated by temperate phages, which may contribute to inflammatory signaling and metabolic dysregulation, though direct causal evidence in humans remains limited (Minot et al., 2011). Although preliminary studies associate diet with gut phage dynamics, direct interventional evidence linking phageome remodeling to metabolic improvements in T2DM remains scarce. While existing literature heavily focuses on Western diet-induced phage alterations as a driver of T2DM pathogenesis (Schulfer et al., 2020; Howard et al., 2024), low-fat dietary interventions offer a compelling, beneficial counterstrategy. Supporting this, mouse fecal virome transplantation demonstrates that low-fat diet-derived viromes significantly ameliorate glucose intolerance and metabolic phenotypes (Rasmussen et al., 2020). Moreover, fiber-rich dietary patterns may help preserve the ecological balance between lytic and lysogenic states and support microbial stability, thereby contributing to gut and metabolic health (Boling et al., 2020; Howard et al., 2024) (Figure 2). Therefore, targeting the diet–phage relationship could become a promising nutritional approach for preventing and managing T2DM, providing a new mechanistic foundation for microbiome-based metabolic interventions (Figure 2). However, most current studies focus primarily on bacterial responses, whereas the contribution of phage dynamics to diet-induced metabolic benefits remains largely unexplored. Therefore, targeting the diet–phage relationship could become a promising nutritional approach for preventing and managing T2DM, providing a new mechanistic foundation for microbiome-based metabolic interventions. Future studies integrating dietary interventions, virome profiling, and metabolic phenotyping will be required to clarify these relationships. However, whether specific dietary patterns exert their beneficial metabolic effects through direct modulation of the gut virome in humans remains to be systematically investigated.

### 4.2 Additional host and environmental factors influencing virome interpretation

In addition to dietary pressure, several host-related factors may further complicate the interpretation of gut virome alterations in patients with T2DM. Obesity and elevated body mass index (BMI) commonly coexist with T2DM and are themselves associated with substantial ecological changes in the gut microbiome and virome. Chronic low-grade inflammation, increased intestinal permeability, altered bile acid composition, and oxidative stress associated with adipose tissue accumulation may together create conditions that favor prophage induction and the expansion of temperate phages (Henrot and Petit, 2022). Obesity has also been linked to reduced microbial gene richness and lower bacterial diversity, which may narrow the range of available bacterial hosts and contribute to the reduced phage α-diversity frequently observed in T2DM cohorts. These observations suggest that some virome features attributed to T2DM may instead reflect broader obesity-related ecological stress rather than diabetes-specific alterations alone.

Aging may also contribute to virome remodeling through several interconnected mechanisms. Because many T2DM cohorts are predominantly composed of older adults, age-related ecological effects can be difficult to separate from diabetes-associated changes. A study published by Ann C Gregory and colleagues in 2020 described how viral diversity changes across the human lifespan (Gregory et al., 2020). In general, viral richness appeared to follow trends broadly similar to bacterial richness in that study, although marked deviations were observed during infancy and the evidence base across the lifespan remains limited and heterogeneous; the relationship between viral and bacterial diversity across aging has not been firmly established (Gregory et al., 2020). This pattern may be related to the immature immune system and the limited protective effects of commensal bacteria in early life, which could increase susceptibility to viral exposure. In older individuals, one possible explanation is that increasing interspecies bacterial competition over time allows more competitive strains to become dominant, thereby reducing overall bacterial richness in later life (Aleman and Valenzano, 2019). Whether this process directly affects viral richness remains unclear, but the parallel trends between bacterial and viral diversity suggest that both may be influenced. This mechanism may also partly explain the increased abundance of crAssphage in older populations, possibly because *Bacteroides* species, which serve as hosts for crAssphage, gain ecological advantages with aging. In addition, aging is associated with immunosenescence, chronic low-grade inflammation, impaired mucosal regeneration, and reduced intestinal barrier integrity. These processes may alter the spatial distribution of phages and their exposure to host immunity. Age-related declines in microbial diversity may further reduce ecological resilience and increase susceptibility to prophage induction under metabolic stress (Shkoporov et al., 2018b). Taken together, some phage alterations observed in elderly patients with T2DM may reflect broader deterioration of mucosal and microbial homeostasis associated with aging, rather than metabolic disease alone.

Drug exposure represents another major source of ecological modulation. Metformin, the first-line therapy for T2DM, has been shown to substantially alter gut microbial composition and metabolic activity. It can increase the abundance of taxa such as Akkermansia muciniphila and *Escherichia* spp. (Wu et al., 2017; Pavlo et al., 2023), while also influencing SCFA metabolism and bile acid signaling. Because phage dynamics are closely linked to bacterial hosts, these drug-induced bacterial shifts may secondarily affect phage populations and phage–host interaction patterns (Wu et al., 2017). Experimental studies further suggest that bacterial metabolic stress caused by altered nutrient flux and redox balance may promote prophage activation through the SOS response pathway, providing a potential mechanistic link between metformin-associated metabolic remodeling and virome changes (Henrot and Petit, 2022; Wu et al., 2017). Similarly, antibiotics are well-recognized inducers of prophage activation. Antibiotic-induced DNA damage and bacterial stress responses can stimulate prophage excision and lytic replication (Wagner and Waldor, 2002; Sutcliffe et al., 2021). Studies in bacterial isolates, together with clinical observations, suggest that antibiotic-induced activation of the RecA protein can trigger the induction of resident prophages. At the same time, antibiotic-associated bacterial depletion may reduce host availability for obligately lytic phages while selectively enriching stress-tolerant lysogenic phages, thereby shifting the ecological balance toward temperate phage dominance.

Overall, current evidence suggests that many virome alterations reported in T2DM may arise from complex interactions among metabolic disease, obesity, aging, drug exposure, diet, and technical variability, rather than from diabetes-specific mechanisms alone. Therefore, future studies should incorporate carefully matched cohorts, longitudinal sampling, medication stratification, and standardized analytical pipelines to better distinguish diabetes-associated virome remodeling from broader signatures of metabolic and environmental dysbiosis.

## 5 Current challenges and therapeutic prospects

### 5.1 Current challenges in gut virome research

Although an increasing number of studies have linked gut virome alterations to T2DM and related metabolic disorders, methodological heterogeneity remains a major barrier to reproducibility and cross-study comparison in virome research. Differences in sample processing, viral enrichment strategies, sequencing approaches, and bioinformatics pipelines can substantially influence estimates of viral diversity, community composition, and phage–host associations, which may in turn lead to inconsistent findings across studies (Shkoporov and Hill, 2019). One of the key sources of variation arises from the use of virus-like particle (VLP) enrichment versus whole-metagenome sequencing strategies. VLP enrichment enhances viral signal and reduces bacterial DNA contamination, but it tends to capture extracellular lytic phages while underrepresenting prophages integrated within bacterial genomes (Shkoporov et al., 2018a; Conceição-Neto et al., 2015; Arumugam et al., 2011). In contrast, whole-metagenome sequencing facilitates the detection of prophages but is more susceptible to host DNA contamination and may increase the risk of viral misclassification (Shkoporov et al., 2018a; Enault et al., 2017). In addition, low-input DNA amplification methods such as multiple displacement amplification (MDA) may preferentially enrich small circular single-stranded DNA viruses, potentially overestimating the abundance of ssDNA phages such as Microviridae (Norman et al., 2015).

Bioinformatics pipelines are another important source of variability. Different viral identification tools, including VirSorter, VIBRANT, and DeepVirFinder, rely on distinct algorithms and reference databases (Gregory et al., 2020; Shkoporov and Hill, 2019). As a result, they often recover partially non-overlapping viral contigs and may generate divergent predictions of AMGs. Moreover, a large proportion of gut viral sequences remain unannotated as “viral dark matter,” and differences among reference databases (e.g., RefSeq Viral, IMG/VR, and the Gut Virome Database) can further affect viral classification, community profiling, and functional annotation. Some studies have also suggested that bacterial DNA contamination may lead to misassignment of metabolic or antimicrobial resistance genes to viral contigs, thereby inflating the inferred functional potential of the virome (Enault et al., 2017). In addition, the inference of phage–host relationships still lack a standardized framework. Methods such as CRISPR spacer matching, sequence homology, co-abundance analysis, and machine learning-based prediction each have inherent limitations. This is particularly relevant for uncultivated phages, where host assignment results are often inconsistent across approaches (Edwards et al., 2016). Therefore, reported associations between specific phages and bacterial taxa in T2DM, such as increased Enterobacteriaceae-associated phages or decreased Bifidobacterium-associated phages, should be interpreted with caution.

Overall, inconsistencies in T2DM virome studies likely reflect not only biological heterogeneity related to diet, obesity, age, and medication exposure, but also the lack of standardization in virome analytical workflows. Future studies should adopt more harmonized pipelines and integrate longitudinal cohorts, multi-omics approaches, and experimental validation to improve reproducibility and advance the field toward mechanistic understanding and clinical translation.

### 5.2 Therapeutic and interventional prospects

Emerging studies suggest that bacteriophage-based strategies, such as phage therapy and phage-rich FVT, may offer new ways to restore microbial balance, improve metabolism, and reduce inflammation in people with T2DM. By selectively removing harmful bacteria or helping phages and bacteria return to a healthy balance, these approaches could open up new possibilities for managing metabolic disorders. In a diet-induced obesity mouse model, transferring the virome from lean donors reduced weight gain and improved glucose tolerance. These benefits were linked to changes in gut microbes and normalized gene expression in the liver and intestine, including genes related to energy use and inflammation, such as Lepr, Ffar2, and Ppargc1a (Rasmussen et al., 2020; Mao et al., 2024).

Animal and human studies have also shown that phage-based interventions can improve metabolism and help restore gut balance. In one experiment, oral administration of an MS2–P22 phage mixture for 7 weeks lowered blood glucose and HOMA-IR levels, increased QUICKI scores, and improved lipid profiles in diabetic mice, with lower TC, TG, and LDL-C and higher HDL-C (Ye et al., 2023/12). These results suggest that phages may help reduce insulin resistance and improve lipid metabolism. Interestingly, these benefits were absent in antibiotic-pretreated mice, suggesting that the effects may depend on the presence of a functional gut microbiota, though this single experimental condition does not exclude alternative explanations such as antibiotic-induced metabolic or inflammatory changes. In humans, an exploratory trial in people with metabolic syndrome found that FMT from lean donors changed the recipients’ gut virome (Manrique et al., 2021), making it more like that of the donors. Further analysis identified 22 phage groups linked to clinical improvement, with 10 enriched in those who responded well. Two phage groups, HV39 and HV84, were positively associated with better insulin sensitivity, measured by glucose disposal rate (Manrique et al., 2021). However, these phage markers still cannot accurately predict individual treatment response. Overall, current evidence supports a role for phages in shaping host metabolism. Because FVT introduces only viral particles, without live bacteria, it may be safer and easier to standardize than traditional microbiome therapies. Still, larger clinical trials are needed to confirm these findings.

Engineered phages also provide a flexible platform for targeting T2DM and metabolic inflammation. Chronic inflammation, impaired insulin signaling, and gut barrier dysfunction are key features of T2DM (Saltiel, 2021; Liang et al., 2023). Recent studies show that genetically modified phages can be programmed to deliver anti-inflammatory or metabolic regulators directly to target bacteria (Pires et al., 2016). For example, Baker and colleagues engineered a T4 phage to produce the serine protease inhibitor Serpin B1a when infecting E. coli K-12 (Baker et al., 2025). This phage reduced neutrophil elastase activity and increased the anti-inflammatory molecule CD24 on neutrophils, effectively easing gut inflammation. In another study, the same group created a T4 phage expressing a ClpB protein that mimics α-MSH. In obese mice fed a high-fat diet, this engineered phage increased satiety hormones such as PYY and GLP-1, reduced food intake and body weight, and lowered inflammatory cytokines like IL-1α, IL-1β, and IL-23 (Baker et al., 2025; Dominique et al., 2021). Similarly, Alexander and colleagues used a natural prophage in *L. reuteri* that can be precisely activated to cause bacterial lysis, resulting in a marked rise in gut levels of therapeutic proteins such as leptin (Alexander et al., 2019). Although *in vivo* effects were not tested, this finding suggests that similar strategies could be used to deliver GLP-1–related or antioxidant proteins to improve insulin sensitivity and energy metabolism. These phage-based delivery systems highlight the promise of phages as programmable tools for precise metabolic therapy. In addition, regulating prophage activation, for example, by using antioxidants or natural compounds to prevent unwanted lytic activity, might reduce LPS release and systemic inflammation, helping restore metabolic balance.

Finally, phage metagenomics may offer new diagnostic insights for T2DM. Metagenomic studies show that certain phage types can clearly distinguish people with T2DM from healthy individuals. Phages that infect *Escherichia* or *Bacteroides* are often more abundant, while those targeting *Lactococcus* are reduced, and these patterns correlate with blood glucose, CRP, and insulin resistance (Chen et al., 2020). When viral and bacterial signals are combined, some studies have reported strong discriminatory performance for identifying T2DM and related complications such as diabetic nephropathy, with AUC values approaching 0.99 in specific study cohorts; these results should be treated as preliminary, as noted above (Fan et al., 2023; Chen et al., 2020; Kirk et al., 2024). However, whether this level of accuracy can be maintained across independent populations remains unclear. This suggests that phages might serve as hidden metabolic markers that help assess disease risk and guide personalized treatment.

Still, clinical evidence remains limited, and the long-term safety and effectiveness of phage-based therapies need to be confirmed. Much remains to be learned about the complex metabolism of the gut microbiome. Even so, using phages to selectively target or silence specific bacteria could become a promising way to reshape microbial metabolism and improve metabolic health in T2DM.

## 6 Conclusion and future directions

Recent findings have revealed that bacteriophages, long overlooked in gut research, play an active part in shaping host metabolism. In T2DM, the gut virome shows reduced diversity, increased temperate phage activity, and functional reprogramming that coincides with metabolic and inflammatory imbalance. These alterations suggest that phages are not merely bystanders responding to microbial stress but may function as active contributors to phage-associated metabolic dysregulation, although causal directionality has not yet been established by current evidence. Through pathways involving ecological restructuring, gene transfer, and immune activation, phages could influence key processes in glucose and lipid metabolism as well as chronic inflammation.

Although progress has been made, much of what we know about phages in T2DM still comes from observation rather than direct evidence. Several important gaps remain. Most viral genes in the gut have unknown functions, so we still cannot tell which ones are involved in metabolism. The causal relationship between changes in phages and metabolic outcomes has not been clearly proven, because most studies use cross-sectional data and lack time-series or experimental validation. In addition, substantial methodological heterogeneity exists across current virome studies, including differences in viral enrichment procedures, sequencing strategies, bioinformatic pipelines, and taxonomic annotation frameworks. These inconsistencies can significantly influence estimates of viral diversity, phage abundance, and host assignment, thereby limiting reproducibility and cross-study comparability. Finally, experimental systems capable of directly interrogating phage–bacteria–host interactions remain limited.

Future research needs to move from description to mechanism and from correlation to explanation. Studies that follow participants over time and combine multiple layers of data, such as viromics, transcriptomics, metabolomics, and immune profiling, will help reveal how specific phage–bacteria relationships affect glucose and lipid metabolism. Experimental systems like organoid cultures, germ-free mice, and synthetic microbial communities can be used to confirm these mechanisms under controlled conditions. Computational tools that use artificial intelligence may also help predict phage–host pairs and clarify the roles of unknown viral genes. At the same time, translational studies should begin exploring precision interventions using phages, including engineered phages, controlled prophage activation, or phage-enriched fecal virome transplantation, to help rebalance gut microbes and reduce inflammation in T2DM.

Ultimately, focusing on phage-associated metabolic dysregulation reframes how we understand the virome’s role in T2DM. Phages should be regarded as active participants in metabolic control rather than passive components of microbial communities. Bridging metagenomic discovery with mechanistic studies, preclinical investigation, and rigorous clinical validation will be essential for translating microbiome-based findings into effective strategies for preventing and treating T2DM and related metabolic disorders.
