# Supplementary material for: Beyond bacterial dysbiosis: the emerging role of gut bacteriophages in type 2 diabetes
Source: Front Cell Dev Biol. 2026 Aug 24;14:1735631. doi: 10.3389/fcell.2026.1735631 (PMC13547728; doi:10.3389/fcell.2026.1735631)

Supplementary Figure 1

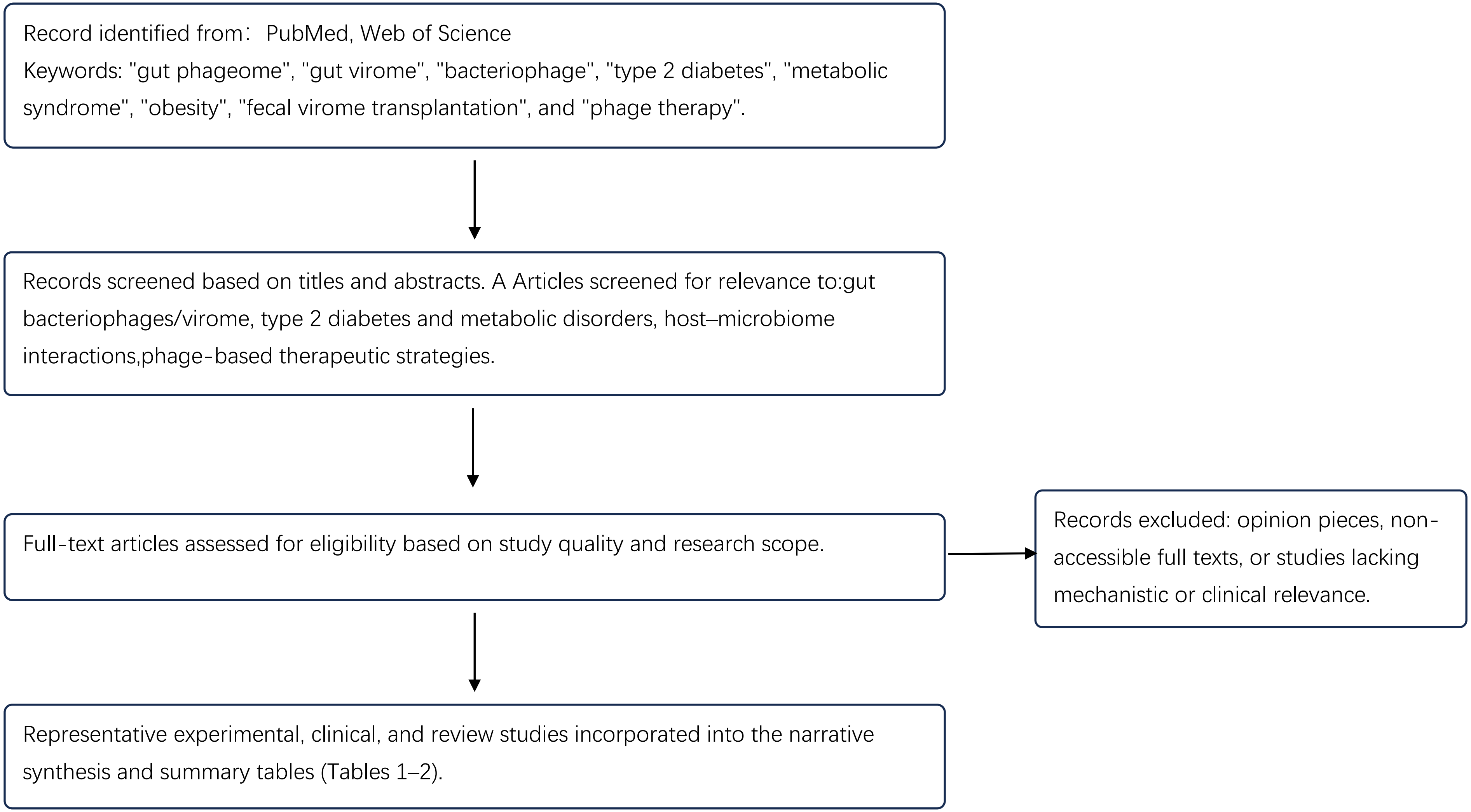

Supplement: Supplementary file 1 [file Supplementaryfile1.pdf]
